# Ubiquitin Carboxy-Terminal Hydrolase L1 (UCH-L1) is increased in cerebrospinal fluid and plasma of patients after epileptic seizure

**DOI:** 10.1186/1471-2377-12-85

**Published:** 2012-08-29

**Authors:** Stefania Mondello, Johanna Palmio, Jackson Streeter, Ronald L Hayes, Jukka Peltola, Andreas Jeromin

**Affiliations:** 1 Banyan Biomarkers, Inc., 113400 Progress Blvd, Alachua, FL, 32615, USA; 2 Department of Neurology, Tampere University Hospital, P.O.Box 2000, Tampere, FIN-33521, Finland; 3 Clinical Department, Banyan Biomarkers Inc., 12085 Research Dr, Alachua, FL, 32615, USA

**Keywords:** Biomarkers, UCH-L1, Epileptic seizures, Neuronal damage

## Abstract

**Background:**

Clinical and experimental studies have demonstrated that seizures can cause molecular and cellular responses resulting in neuronal damage. At present, there are no valid tests for assessing organic damage to the brain associated with seizure. The aim of this study was to investigate cerebrospinal fluid (CSF) and plasma concentrations of Ubiquitin carboxy-terminal hydrolase L1 (UCH-L1), a sensitive indicator of acute injury to brain neurons, in patients with tonic–clonic or partial secondarily generalized seizures due to various etiologies.

**Methods:**

CSF and plasma concentrations of UCH-L1 were assessed in 52 patients within 48 hours after epileptic seizure and in 19 controls using ELISA assays.

**Results:**

CSF obtained within 48 hours after seizure or status epilepticus (SE) presented significantly higher levels of UCH-L1 compared to controls (p = 0.008). Plasma UCH-L1 concentrations were negatively correlated with time to sample withdrawal. An analysis conducted using only the first 12 hours post-seizure revealed significant differences between concentrations of UCH-L1 in plasma and controls (p = 0.025). CSF and plasma concentrations were strongly correlated with age in patients with seizure, but not in control patients. Plasma UCH-L1 levels were also significantly higher in patients after recurrent seizures (n = 4) than in those after one or two seizures (p = 0.013 and p = 0.024, respectively).

**Conclusion:**

Our results suggest that determining levels of neuronal proteins may provide valuable information on the assessment of brain damage following seizure. These data might allow clinicians to make more accurate therapeutic decisions, to identify patients at risk of progression and, ultimately, to provide new opportunities for monitoring therapy and targeted therapeutic interventions.

## Background

Epilepsy is a common acquired chronic neurological disorder occurring in ~1% of the general population. Recent experimental studies have shown that seizures, whether continuous or sporadic, can cause molecular and cellular responses resulting in neuronal damage or death, gliosis, and axonal and dendritic remodeling [1,2]. Although some efforts have been made to identify biochemical markers in CSF or blood of patients with epileptic seizure that could assess organic damage or aid in the diagnosis and monitoring of disease progression, to date, none of those markers has gained clinical importance [1,3,4].

Ubiquitin C-terminal hydrolase (UCH-L1) is a neuron-specific cytoplasmic enzyme, highly enriched in neurons [5]. Increased cerebrospinal fluid (CSF) and blood concentrations of UCH-L1 have been associated with processes of neuron destruction (loss) and increased blood brain barrier (BBB) permeability [6]. UCH-L1 concentration has been reported to be elevated in a number of neurological diseases including aneurysmal subarachnoid hemorrhage, traumatic brain injury (TBI), stroke and neonatal hypoxic-ischemic encephalopathy (HIE) [7-10]. After TBI, blood UCH-L1 level correlates with injury severity, outcome at discharge and 6 months after injury [10,11]. Remarkably, this neuronal protein can be readily detected in CSF and blood very early after injury [11], which provides a valuable time-window for potential neuroprotective strategies. Because of its clinically relevant characteristics and high brain specificity, UCH-L1 has been recently recognized as a novel and promising biomarker of neuronal injury and BBB disruption.

To our knowledge, no studies have assessed UCH-L1 in patients after seizures. Therefore, the objective of the present investigation was to evaluate CSF and plasma concentrations of UCH-L1 in patients with tonic–clonic or partial secondarily generalized seizures and their relationship to clinical characteristics including etiology and number of seizures.

## Methods

### Subjects

This study enrolled a total of 52 patients with single or recurrent tonic–clonic or partial secondarily generalized seizures admitted at the Emergency Department of Neurology, Tampere University Hospital, Finland. The study protocol was approved by the Ethics Committee of Tampere University Hospital. Written informed consent was obtained from all patients. This investigation was part of an ongoing study aimed to assess biochemical markers in patients with epileptic seizures. Forty-eight of the patients evaluated in the present study and their biochemical analyses have been previously reported elsewhere [4].

Patients were classified according to the etiology of epileptic seizures into alcohol withdrawal group (n = 15), acute systemic illness or acute CNS disorder group (n = 6), remote symptomatic epilepsy group (n = 14) and cryptogenic focal or idiopathic epilepsy group (n = 17). Patient demographics and clinical characteristics are given in Table1. The etiology of acute systemic illness or acute CNS disorder included sepsis, hyponatremia, acute brain infarction, encephalitis and necrotizing encephalomyelitis. The etiology of remote symptomatic epilepsy included brain tumor (n = 5), post-stroke or post-traumatic epilepsy (n = 6), Alzheimer’s disease (n = 1), multiple sclerosis (n = 1), and post-encephalitic epilepsy (n = 1). Patients with cryptogenic focal or idiopathic epilepsy did not present any abnormal finding on computed tomography (CT) or magnetic resonance imaging (MRI) of the brain. CSF and venous blood samples were taken within 48 h after the seizure (median 12.5 hrs).

**Table 1:** Demographic and clinical characteristics of patients with epileptic seizure and controls

**Characteristic**	**Epileptic Group (n = 52)**	**Controls (n = 19)**	**P value**
**Age, years, median (IQR)**	45.5 (31.5-62)	41 (32–45)	0.13
**Male, n (%)**	30 (57.69)	4 (21.05)	0.014*
**Time to sampling, h**	12.5 (7–20)	-	
**Epilepsy Etiology**			
Alcohol Withdrawal	15 (28.85)	-	
Acute Systemic Illness or Acute CNS Disorder	6 (11.54)	-	
Remote Symptomatic Epilepsy	14 (26.92)	-	
Cryptogenic Focal	14 (26.92)	-	
Idiopathic Epilepsy	3 (5.77)	-	
**Seizure Characteristic**			
One	27 (51.92)	-	
Two	11 (21.15)	-	
Three	1 (1.92)	-	
Four	4 (7.69)	-	
Status Epilepticus	9 (17.31)	-	

Mann–Whitney *U*-test for continuous variables, cross-tabulations and chi-square-test for categorical variables.

We included a control population consisting of 19 individuals without history of central nervous system disease or seizures on whom lumbar was performed to exclude neurological disorders. Neurological examination, neuroimaging (CT or MRI of the head) and laboratory analysis showed normal results for all controls (Table1).

### UCH-L1 determination

Approximately 15 mL of CSF and 5 mL of plasma were collected from each subject. Samples were centrifuged for 10 minutes at 4000 rpm and immediately frozen and stored at −70°C until the time of analysis. All samples were analyzed in duplicate. Samples were measured using a standard UCH-L1 sandwich ELISA protocol as described below. Reaction wells were coated with capture antibody (500 ng/well purified anti-rabbit UCHL1, made in-house) in 0.1 M sodium bicarbonate, pH 9 and incubated overnight at 4°C. Plates were then emptied out and 300 μl/well blocking buffer (Startingblock T20-TBS) was added and incubated for 30 min at ambient temperature with gentle shaking. This was followed by addition of antigen standard (UCHL1 standard curve: 0.05 - 50 ng/well) unknown samples (3–10 uL CSF) or assay internal control samples. The plate was incubated for 2 hours at room temperature then washed using an automatic plate washer (each well rinsed with 5 x 300 μl with wash buffer (TBST)). Detection antibody (anti-rabbit UCH-L1-HRP conjugation, made in-house at 50 μg/mL) in blocking buffer was then added to wells at 100 μl/well and the plates were further incubated for 1.5 hours at room temperature. After additional automatic washing, biotinyl-tyramide solution (Perkin Elmer Elast Amplification Kit) was added and the plate was incubated for 15 minutes at room temperature followed by automatic washing. Addition of Streptavidin-HRP (1:500, 100ul/well) in PBS with 0.02% Tween 20 and 1% BSA for 30 minutes incubation at room temperature preceded automatic washing. Lastly, the wells were developed with substrate solution: Ultra-TMB ELISA 100ul/well (Pierce# 34028) with incubation for 5–30 minutes and read at 652 nm with a 96- well spectrophotometer (Molecular Device Spectramax 190).

### Statistical analysis

All statistical analyses were performed using SAS (SAS version [9.2] of the SAS System. Copyright © 2002–2008 by SAS Institute Inc., Cary, NC, USA). Exploratory analysis was carried out to determine the distribution of the data. Continuous variables are presented as mean (SD) or median (interquartile range), as appropriate. Distributions of categorical variables are presented as frequencies and percentages. Categorical variables were compared using the chi-square or Fisher’s exact test, as appropriate. The Mann–Whitney test was used to compare biomarker concentrations between two groups and the Kruskal-Wallis test was used in case of three or more groups. Spearman rank correlation test was used to test correlations between biomarkers and continuous variables. All hypothesis tests conducted were 2-tailed. A p value < 0.05 was considered significant.

## Results

**Figure 1:**
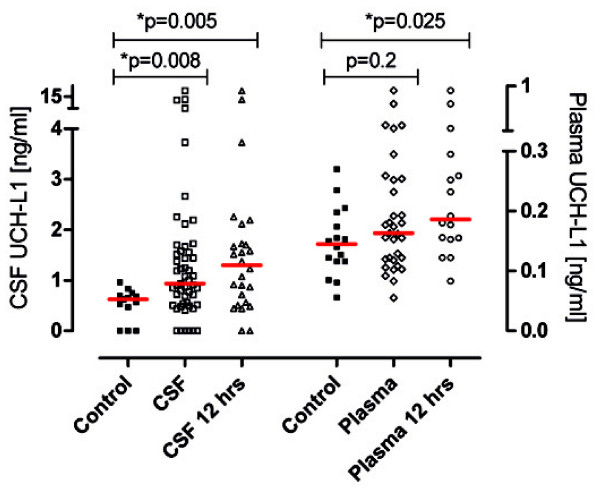
**UCH-L1 concentration in CSF and plasma of patients within 48 hrs and within 12 hrs after seizure and in controls.** The red horizontal line represents the median. Significant differences are indicated with * (P < .05) (Mann–Whitney U-test).

A total of 52 patients and 19 controls were included for analyses. As indicated in Table1, the characteristics of patients and controls were similar except for the gender: 58% of subjects with seizure compared with 21% of controls were male. UCH-L1 concentrations in CSF were significantly higher in patients within 48 hrs after epileptic seizure than in controls. Plasma UCH-L1 concentrations were similar in both study groups. The median value and interquartile range of UCH-L1 for patients with epileptic seizure and for controls are shown in Table2 (Figure1). Furthermore, plasma UCH-L1 concentrations were negatively correlated with time to sample withdrawal (R = −0.38, P =0.029), but no correlations with UCH-L1 in CSF were found. An analysis conducted using only the first 12 hours post-seizure showed significant differences between concentrations of UCH-L1 in plasma and control group (p =0.025) as well as in CSF and controls (p = 0.005) (Table2, Figure1). 

**Table 2:** CSF and plasma concentration of UCH-L1 in patients with seizure and in controls

		**N**	**Epileptic Group (n = 52)**	**N**	**Controls (n = 19)**	**P-value**
**After seizure**	**CSF UCH-L1**	51	0.934 (0.513-1.582)	13	0.627 (0.234-0.723)	0.008*
	**Plasma UCH-L1**	33	0.163 (0.119-0.253)	16	0.145 (0.116-0.192)	0.2
**Within 12 hrs after seizure**	**CSF UCH-L1**	26	1.300 (0.55-1.822)			0.005*
	**Plasma UCH-L1**	16	0.186 (0.154-0.286)			0.025†

Data are given as median (interquartile range).

* *p <* 0.01, † *p <* 0.05 (*p* values of the Mann–Whitney test for differences between the groups [patients versus controls]).

**Figure 2:**
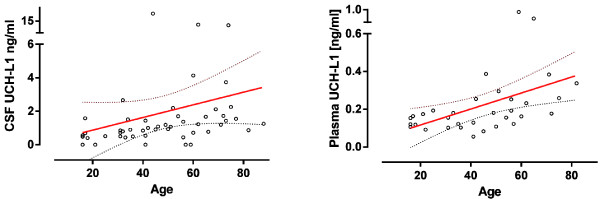
**UCH-L1 concentration in CSF and plasma and age in patients with epileptic seizure.** Biomarkers were positively correlated with age in patients with epileptic seizure. Linear regression line and 95% CI were given (on the right) (n = 51).

CSF and plasma levels of UCH-L1 were not affected by sex. UCH-L1 in CSF and plasma were strongly correlated with age in patients with seizure, but not in controls (Figure2, Table3). Furthermore, CSF UCH-L1 concentrations were positively correlated with plasma concentrations (R = 0.38, P =0.030). 

**Table 3:** Spearman correlation coefficient (p value) between biomarker concentrations and demographic variable

	**Age**	
	**Patients with seizures**	**Controls**
**CSF UCH-L1**	0.70 (<.0001)*	0.21 (0.74)
**Plasma UCH-L1**	0.55 (0.0025)*	−0.6 (0.28)

**Figure 3:**
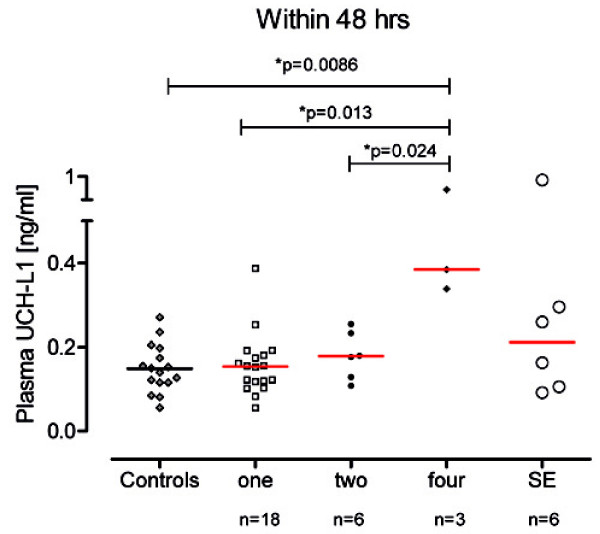
**Plasma UCH-L1 concentration in patients within 48 hrs after single or recurrent seizures, or status epilepticus.** The red horizontal line represents the median. Significant differences are indicated with * (P < .05) (Mann–Whitney U-test).

The number of seizures showed significant differences also for concentration of UCH-L1 in plasma. Patients with recurrent seizures (n = 4) had significantly higher plasma UCH-L1 concentrations than patients with one or two seizures (median, 0.38 vs 0.15 vs 0.18 ng/ml, p = 0.013 and p = 0.024, respectively) (Figure3). CSF UCH-L1 concentrations did not differ between these subpopulations, but a trend of increasing number of seizures was observed (data not shown).

## Discussion

A series of experimental studies from multiple laboratories have provided evidence for the association of epilepsy with brain damage and have shown that seizures can induce neuronal loss [12-14]. These findings were also confirmed in humans. In particular, previous studies examining brain tissue resected for the treatment of drug-refractory epilepsy have indicated that recurrent generalized seizures and long duration of epilepsy are associated with severe neuronal loss [15-18]. Seizure-induced neuronal death has been reported to result primarily from excitotoxicity that leads to DNA damage and protease activation, culminating in necrosis [19], but activation of programmed (apoptotic) cell death pathways following seizures has also been found [20,21]. Biochemical markers identifying neuronal damage or loss associated with injury have been sought. Increased levels of neuron-specific enolase (NSE), a marker of neuronal damage, has been demonstrated in CSF and serum of both children and adult patients after spontaneous and evoked epileptic seizures, but the findings were not consistent and very few risk factors for increased NSE levels have been identified [3,22-28].

UCH-L1 has previously been identified as neuronal cell body injury by our and others groups in a number of different neurological conditions [7-11,29]. The focus of the current study was to evaluate the concentration of UCH-L1 in CSF and plasma of patients in the acute phase after epileptic seizures (median interval between epileptic seizure and sample collection 12.5 h).

We found that elevated UCH-L1 concentrations occur in CSF of patients within 48 hrs after epileptic seizure. In addition, our study showed that increased plasma UCH-L1 concentrations are found within 12 h but not within 48 after epileptic seizure. This observation has 2 major implications. First, UCH-L1 seems to detect damage at a very early time point after seizure when potential neuroprotective strategies might be most effective. Second, this finding might be explained by the fact that elevated plasma UCH-L1 reflects disruption of the BBB during the acute phase after seizure. Indeed, previous reports have suggested that prolonged seizure activity in animals and humans, as well as the occurrence of brief, induced, and spontaneous seizures, may be associated with opening of the BBB [30-32]. This consideration is also supported by the negative correlation between UCH-L1 plasma concentrations and time to sample withdrawal.

Remarkably, patients with recurrent seizures had higher UCH-L1 levels in plasma than patients with single event and UCH-L1 levels increased with the number of seizures (Figure3). Supporting these observations, a number of experimental studies have provided compelling evidence of an association between recurrent seizures and neuronal loss in animals [13-15,33] and histological analysis has indicated that as the number of seizures increases, the damage becomes more severe [13,14]. In humans, histological findings [16,18,34] as well as sensitive imaging techniques have provided evidence that seizure frequency is associated with the severity of structural brain damage [35-38]. Kälviäinen et al. [36], however, suggest that a large number of seizures are needed to result in structural damage detectable by neuroimaging (MRI), while individual seizures cause relatively limited damage involving a small number of neurons that may be easily overlooked or remain undetected. Therefore, the detection of damage caused by a single seizure *in vivo* challenges the resolution of anatomical or imaging techniques. As a consequence, the cumulative seizure-induced structural damage resulting from subtle but recurring insults may take decades before becoming evident along with the slowly evolving cognitive decline associated with the damage which may be difficult to detect in clinical practice until an advanced status. Therefore, biochemical markers such as UCH-L1 are necessary and may represent an extremely sensitive method for the assessment of mild damage following seizure and a simple but critical way to monitor the progression of the disorder.

Two important promising characteristics of UCH-L1 should be noted: the half-life (ranging from 7 to 9 hours, in CSF and serum, respectively) [11] and a prompt detection soon after injury (within 3 hours, in our study). Indeed, the rapid appearance associated with a rapid elimination allows assessment of the amount of seizure-related brain damage and, by using repeated measurements, makes it possible to distinguish between this injury and other chronic and underlying conditions or secondary injuries. Furthermore, when the assay is ported to a point-of-care (POC) system, accurate results will be available to the clinician with a short turnaround time (within 30 min or less), [39] allowing rapid ED assessment.

Additionally, the increased UCH-L1 levels observed after recurrent seizure may reflect the continuing molecular, cellular, or network changes and neuronal reorganization associated with the recurring and cumulative damage responsible for the long-term alterations in neural circuits and progression of the disorder.

Interestingly, CSF and plasma UCH-L1 concentrations showed a strong correlation with age in patients with seizures but no correlation was found in controls. Together, these data suggest that age may influence the severity of seizure-related damage and, therefore, the release of brain-specific markers into the CSF and through the BBB into the circulation. Consistently, previous studies did not find neuronal loss when generalized seizures were induced in immature animals suggesting an early developmental resistance to seizure-induced damage [40-43]. An age-dependent increase in brain damage after seizures was also reported in humans [17]. Nevertheless, it is important to note that previous studies have shown that, even in the absence of overt neuronal injury, immature brain undergoes neuronal reorganization after recurrent seizures with mossy fiber sprouting, asymmetric synapses and neurogenesis [42,43]. In addition, prolonged seizures have been shown occasionally to produce acute injury in children that has evolved to hippocampal atrophy [44]. Therefore, for clinical use, including the pediatric setting, a multimarker strategy could be the most promising and accurate approach in refining risk stratification among children with acute epileptic seizures by providing information on the cell type and subcellular localization of injury as well as on the biochemical pathways and underlying physiology of cell death that can result in progression of the disorder.

Our study has several limitations. No accurate data were available regarding seizure duration. The time interval between seizure, lumbar puncture and plasma sampling varied. However, the sampling was performed at the earliest time point available after admission and not more than 48 hrs after seizure. Furthermore, our cohort represents heterogeneous etiologies of epileptic seizures. These variables may reflect different features of the underlying disease process and its damaging effects. We emphasize that further studies addressing the relationship between neuronal damage and BBB disruption following seizure are needed. However, the intent of this exploratory study was to investigate the potential role of UCH-L1 as a putative biomarker of neuronal injury and BBB dysfunction in patients after seizure in an emergency department, where this information could be critical to improve the management and guide a timely and effective treatment.

## Conclusion

This study presents the first results of increased CSF and plasma concentrations of UCH-L1 in patients after seizures. Our data may stimulate further research to evaluate the utility of UCH-L1 as a surrogate marker for brain damage in epilepsy. We believe that the identification of CNS-specific proteins, providing important insight into underlying mechanisms and damage following seizure, could support the choice of timely and effective treatments tailored to the clinical situation and reveal new opportunities for intervention and drug development.

## Abbreviations

UCH-L1: Ubiquitin carboxy-terminal hydrolase l1; CSF: Cerebrospinal fluid; TBI: Traumatic brain injury; BBB: Blood brain barrier; SE: Status epilepticus; HIE: Hypoxic-ischemic encephalopathy; NSE: Neuron-specific enolase; MRI: Magnetic resonance imaging; CNS: Central nervous system.

## Competing interests

Drs. Stefania Mondello and Andreas Jeromin are employees and received salaries from Banyan Biomarkers, Inc. Drs. Streeter and Hayes own stock, receive royalties and salaries from, and are officers of Banyan Biomarkers Inc., and as such may benefit financially as a result of the outcomes of this research or work reported in this publication. Banyan Biomarkers, Inc. filled patent applications based upon the disclosure of this publication. Drs. Palmio and Peltola have no conflicts of interest.

## Authors’ contributions

SM performed the statistical analysis, contributed to interpretation of the results and drafted the manuscript. AJ participated in the laboratory work and in manuscript preparation. JP and JP contributed to the study’s design, data collection and manuscript preparation. JS and RLH contributed to manuscript editing. All authors have read and approved the article for publication.

## Pre-publication history

The pre-publication history for this paper can be accessed here:

http://www.biomedcentral.com/1471-2377/12/85/prepub
